# Long Non-coding RNAs in Hepatitis C Virus-Infected Cells

**DOI:** 10.3389/fmicb.2017.01833

**Published:** 2017-09-28

**Authors:** Marina Barriocanal, Puri Fortes

**Affiliations:** Department of Gene Therapy and Hepatology, Navarra Institute for Health Research (IdiSNA), Centro de Investigación Médica Aplicada, University of Navarra, Pamplona, Spain

**Keywords:** lncRNAs, HCV, proviral, antiviral, IFN response, liver cirrhosis, fibrosis, HCC

## Abstract

Hepatitis C virus (HCV) often leads to a chronic infection in the liver that may progress to steatosis, fibrosis, cirrhosis, and hepatocellular carcinoma (HCC). Several viral and cellular factors are required for a productive infection and for the development of liver disease. Some of these are long non-coding RNAs (lncRNAs) deregulated in infected cells. After HCV infection, the sequence and the structure of the viral RNA genome are sensed to activate interferon (IFN) synthesis and signaling pathways. These antiviral pathways regulate transcription of several cellular lncRNAs. Some of these are also deregulated in response to viral replication. Certain viral proteins and/or viral replication can activate transcription factors such as MYC, SP1, NRF2, or HIF1α that modulate the expression of additional cellular lncRNAs. Interestingly, several lncRNAs deregulated in HCV-infected cells described so far play proviral or antiviral functions by acting as positive or negative regulators of the IFN system, while others help in the development of liver cirrhosis and HCC. The study of the structure and mechanism of action of these lncRNAs may aid in the development of novel strategies to treat infectious and immune pathologies and liver diseases such as cirrhosis and HCC.

## Hepatitis C Virus (HCV) Infection

**FIGURE 1:**
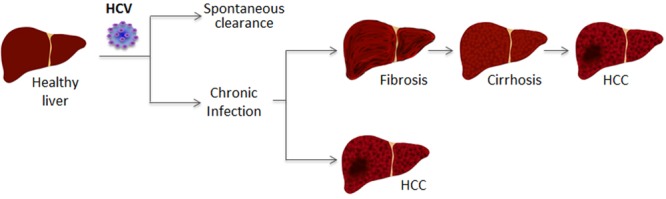
Schematic of liver disease in HCV-infected patients.

Hepatitis C virus is a hepatotropic virus member of the Flaviviridae family discovered almost 30 years ago (Choo et al., 1989, 1991). HCV infection affects 1.6% of the world’s population (≌115 million people) with a prevalence of 0.1–23% depending on the region (Gower et al., 2014; Daw et al., 2016). Asia has the highest prevalence, with 60% of the HCV-infected people. Out of the six major genotypes described for HCV, the most common is genotype 1, which is responsible for 46% of all infections, followed by genotypes 3, 2, 4, and 6. HCV infection is mainly caused by iatrogenic transmission in low- and middle-income countries, and by risky sexual practices and sharing needles while tattooing or injecting drugs in developed countries (Lanini et al., 2016). After HCV entry, the virus produces an acute infection which spontaneously resolves in 15–50% of cases within the first 12–16 weeks, but which may become chronic in 55–85% of the cases (Ghany et al., 2009; AASLD/IDSA HCV Guidance Panel, 2015) (**Figure 1**). In many cases, the infection remains undiagnosed for many years, as chronic infection is frequently asymptomatic. The infection causes a liver injury that may progress to liver steatosis, fibrosis, cirrhosis and eventually hepatocellular carcinoma (HCC) (Carnero and Fortes, 2016; Miao et al., 2017) (**Figure 1**). Therefore, it is important to detect chronic HCV infection before the development of liver disease. Detection methods include identification of HCV RNA by PCR and HCV proteins by ELISA (Chevaliez and Pawlotsky, 2008; Ghany et al., 2009; AASLD/IDSA HCV Guidance Panel, 2015). There is no vaccine for prevention of HCV infection but direct antivirals have recently been developed that have attained sustained virological response rates higher than 90% (Poordad and Khungar, 2011; Sarrazin et al., 2012; Sheridan et al., 2013; Bhamidimarri et al., 2017; Cento et al., 2017). However, given the prevalence of the infection and the high mutation rate of HCV, which helps the virus to generate escape mutants resistant to the inhibitors, a strict epidemiological vigilance and a dedicated scientific research into HCV-infection should not stop (Perales et al., 2015; Kan et al., 2017; Raj et al., 2017; Wyles et al., 2017). Effective vaccines against HCV will be required before the goal of a complete cure for HCV can be achieved.

### The Viral Particle

Hepatitis C virus is a small (40–80 nm in size), enveloped virus that contains the viral genome bound to the core protein (Gastaminza et al., 2010; Catanese et al., 2013). The viral genome is a single-stranded RNA molecule of 9.6 kilobases and positive polarity (Lemon et al., 2007). The genome serves as messenger RNA and consists of a long open reading frame (ORF) flanked by highly structured 3′ and 5′ un-translated regions (UTRs), which are required for viral translation, replication and encapsidation. Interestingly, the 3′ and 5′ ends of HCV interact with each other (Romero-Lopez and Berzal-Herranz, 2009).

The 5′ UTR includes an internal ribosome entry site (IRES) that allows cap-independent translation of a single polyprotein (Sagan et al., 2015; Adams et al., 2017). The polyprotein is approximately 3000 amino acids in length and is co- and post-translationally cleaved by viral and cellular proteases to produce all mature viral proteins: three structural proteins (core, E1, and E2) and seven non-structural proteins (p7, NS2, NS3, NS4A, NS4B, NS5A, and NS5B). Importantly, HCV proteins are characterized by their multi-functionality (Moradpour and Penin, 2013). The core protein is the main component of the HCV nucleocapsid and promotes nucleocapsid assembly (Boulant et al., 2006). The envelope of the viral particle contains the viral E1 and E2 glycoproteins, required for cell attachment and entry and for the assembly of the infectious HCV particle. P7 participates in HCV assembly and release (Moradpour and Penin, 2013; Atoom et al., 2014). NS2 autoprotease is required for the cleavage of NS2/NS3 and participates in virus assembly (Schregel et al., 2009; Jirasko et al., 2010). The replicase complex is formed by NS3, NS4A, NS4B, NS5A, and NS5B. NS3 is another viral protease and helicase. As a protease, NS3 processes non-structural proteins and cleaves some host factors involved in the antiviral response (Moradpour and Penin, 2013). As a helicase, NS3 is crucial for HCV RNA unwinding and replication and for viral particle assembly. NS4A helps binding of NS3 to the endoplasmic reticulum (ER), and increases NS3 activity. NS4B participates in viral assembly and is a key protein for the formation of the membranous web (MW) required for replication (Han et al., 2013). NS5A binds several proteins, the viral RNA and the membranes to participate in MW formation, virus replication and assembly and to block the antiviral response (Moradpour and Penin, 2013; Schoggins and Rice, 2013). Finally, NS5B is the RNA-dependent RNA polymerase essential for viral replication and assembly (Lohmann, 2013). Similar to other RNA polymerases, NS5B lacks proofreading activity. Viral progeny is a collection of viruses with mutated viral genomes known as *quasi-species* with a high capacity to escape the immune host system (Marascio et al., 2014).

### The Viral Cycle

Hepatitis C virus viral particles circulate in the blood stream as lipoviroparticles (LVPs), attached to lipids and apolipoproteins (Apo) from the host (Grassi et al., 2016). This shell facilitates the infection of hepatocytes and may aid in viral escape from neutralizing antibodies. HCV entry into the hepatocytes is a highly regulated multi-step process that involves various cellular proteins. Once the virus is in the liver, at the space of Disse, it is captured by heparan sulfate proteoglycan receptors present in the basolateral membrane of the hepatocytes (Xu et al., 2015). There, LVPs are sequentially hydrolysed, the associated ApoB is uncovered, and ApoB exposure enables low-density lipid receptor (LDLR) binding to the LVPs. Then, these LVPs interact with scavenger receptor class B type I and CD81 (Chang et al., 2017), which allows migration of the particle to tight junctions, binding to claudin 1 (CLDN1) and occludin (OCLN) and internalization by endocytosis. After uncoating driven by the acidification of the endosome, the viral genome is released into the cytosol (Grassi et al., 2016; Miao et al., 2017).

Once the HCV genome is in the cytoplasm, viral replication takes place in the MW, a specialized structure formed by double-membrane vesicles originating from the ER (Grassi et al., 2016). NS5A binds the HCV RNA and the viral RNA polymerase NS5B is in charge of replication. First, the positive-stranded RNA serves as template for the production of intermediate negative-stranded RNAs. Then, the negative-stranded RNAs are copied into new positive-strand RNA viral genomes, which can be used for translation, replication, or packaging (Manns et al., 2017).

Hepatitis C virus viral particle assembly occurs in the ER close to membrane-bound lipid droplets (LDs). NS5A binds the 3′ UTR of positive-stranded HCV RNAs and transfers the genomes from the replication complexes in the MW to the core proteins bound to the ER (Targett-Adams et al., 2010; Shi G. et al., 2016). Then, the nucleocapsids acquire their envelope, most probably, after budding into the ER. The new HCV virions adhere to nascent VLDL or LDL particles and form the LVPs that are transported to the Golgi for virus release (Syed et al., 2017). Therefore, a proper functionality of the lipid secretion pathway and a proper synthesis of VLDLs is essential for HCV release (Bassendine et al., 2013). HCV can be also transmitted directly from cell to cell, escaping the inhibitory action of neutralizing antibodies (Timpe et al., 2007; Brimacombe et al., 2011).

### HCV Host Factors

Hepatitis C virus draws on multiple host cell factors for entry, replication, assembly, and release. As mentioned previously, key factors in the HCV cycle are: (i) host lipoproteins such as ApoE, ApoA, ApoB, or MTP that attach to HCV nucleocapsid and are required for entry (Chang et al., 2007; Huang et al., 2007; Mancone et al., 2012; Fukuhara et al., 2015); (ii) several hepatocyte receptors including CLDN1 and OCLN (Li Q. et al., 2016); (iii) host factors required for MW formation, such as cyclophilin A or kinases like phosphatidylinositol-4-kinase III alpha (PI4KIIIalpha) (Delang et al., 2012; Rosnoblet et al., 2012; Ross-Thriepland et al., 2013; Colpitts et al., 2015); (iv) cellular proteins that attach to the IRES and allow translation, like DDX5 and PARP1 (Ríos-Marco et al., 2016).

All of these are protein factors. However, efficient HCV infection also requires the expression of cellular micro and long non-coding RNAs (miRNAs and lncRNAs). The latter are the focus of this review and deserve a special chapter. In fact, upon HCV infection, the expression of many host miRNAs and lncRNAs is altered (Singaravelu et al., 2014b; Carnero and Fortes, 2016). Surprisingly, the miRNA miR-122 is one of the most limiting factors for HCV viability and development of HCV-associated HCC (Jopling et al., 2005; Kutay et al., 2006). miR122 is a liver-specific microRNA whose levels are upregulated by HCV. miR122 binds two conserved regions located at the 5′ end of the viral genome (Adams et al., 2017). Binding protects viral RNA from degradation by exonucleases, enhances HCV RNA replication, stimulates IRES-mediated translation and hides the 5′ end of the HCV genome from cellular sensors that activate the antiviral response (Henke et al., 2008; Shimakami et al., 2012; Li Y. et al., 2013; Sedano and Sarnow, 2014). This results in increased levels of viral RNA and viral proteins. It is not surprising that HCV RNA levels can be reduced in infected patients with a drug that blocks miR122 (Janssen et al., 2013). Remarkably, sequestration of cellular miR-122 by the viral genome results in increased levels of miR-122-targets and increased oncogenic potential of HCV (Israelow et al., 2014; Luna et al., 2015). Finally, it has been recently reported that miR122 also binds the NS5B coding region and the 3′ UTR, which is essential for HCV replication (Gerresheim et al., 2017). Other miRNAs altered upon HCV infection include miR199a-3p, let7-b or miR-181c, which inhibits replication by binding to E1 and NS5A sequences. miR185-5p and miR-27 are induced by core protein and control genes involved in lipid metabolism (Murakami et al., 2011; Cheng et al., 2012; Shirasaki et al., 2013; Singaravelu et al., 2014a; Li M. et al., 2015; Mukherjee et al., 2015). miR-208b and miR-499a-5p are induced by HCV and target a polymorphic region of interferon lambda (IFNL3) in the 3′ UTR in charge of controlling IFNL3 mRNA stability, decreasing the antiviral response against HCV infection (McFarland et al., 2013).

## The Antiviral Response Against HCV

### Interferon Synthesis Pathway

**FIGURE 2:**
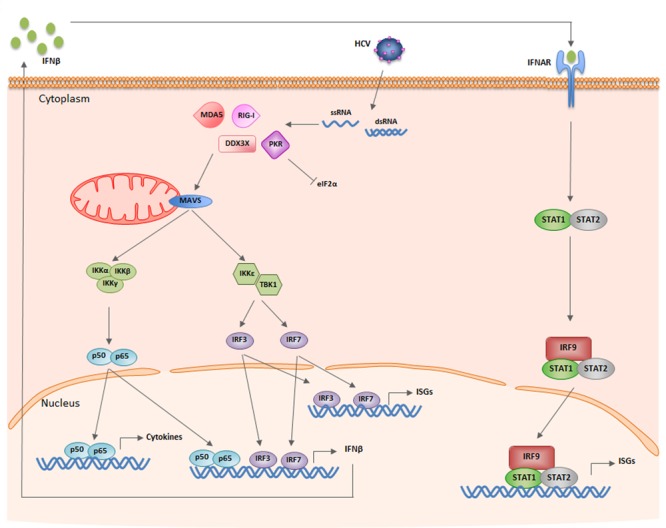
Schematic of the IFN synthesis and signaling pathways induced by HCV infection. See text for details.

The cell has several mechanisms to recognize and fight against exogenous pathogens. Viruses produce pathogen-associated molecular patterns (PAMPs) that can be recognized by PAMP recognition receptors (PRRs). These PRRs are located on the cell surface or in intracellular compartments as endosomes, and identify viral RNA structures not present in the host cell by discriminating between viral RNA and RNA from the host. Toll-like receptors (TLRs) are able to recognize viral nucleic acids both in and out of the cell, while retinoic acid-inducible gene I (RIG-I) and melanoma differentiation-associated gene 5 (MDA5) sense intracellular viral ssRNA or dsRNA, according to the 5′ end structure and length, respectively (Scheel and Rice, 2013) (**Figure 2**). In the case of the HCV genome, the pU/UC tract located in the 3′ UTR is sensed by RIG-I in the cytoplasm of the cell soon after the virus has released its genome (Saito et al., 2008). Further, RIG-I and TLR3 can also sense dsRNA produced by viral replication (Binder et al., 2011; Dabo and Meurs, 2012; Li et al., 2012). RIG-I is auto inhibited by its regulatory domain, and upon sensing viral RNA, RIG-I undergoes conformation changes and ubiquitination by E3 ligase TRIM25 that lead to its dimerization and activation (Gack et al., 2007; Oshiumi et al., 2013b). Then, RIG-I associates with mitochondrial antiviral signaling protein (MAVS) via CARD-CARD (caspase activation and recruitment domains) interactions and this recruits TNF receptor associated factors (TRAFs) (Liu et al., 2012). On one hand, MAVS can activate TBK1/IKK𝜀 kinase (Hemmi et al., 2004). Then, interferon regulatory factors (IRFs) 3 and 7 are phosphorylated by IKK𝜀 and activated. On the other hand, MAVS activates nuclear factor κB (NF-κB) through IKKα/IKKβ/IKKγ (Sharma et al., 2003; Thompson et al., 2011; Sun et al., 2015; Yang and Zhu, 2015). IRFs induce a first wave of interferon (IFN)-stimulated genes (ISG), NF-κB induces inflammatory cytokines and, together, they activate type I IFN synthesis and secretion.

While this is the major IFN synthesis pathway, the HCV genome can also be sensed by non-canonical receptors such as protein kinase R (PKR) and DEAD box RNA helicase 3 (DDX3X) (**Figure 2**). PKR is a cytoplasm protein that binds the dsRNA IRES located at the 5′ UTR of the viral genome in a sequence-independent manner. PKR activation after dsRNA binding triggers interaction of PKR with MAVS, MAVS activation and transcription of ISGs (Dabo and Meurs, 2012). Further, active PKR phosphorylates eukaryotic translation initiation factor eIF2α and impairs translation (Garaigorta and Chisari, 2009) (see below). DDX3X interacts with the 3′ UTR region of HCV and activates IKKα. Unexpectedly, PKR and DDX3X are cellular sensors that favor HCV replication (Ariumi et al., 2007; Garaigorta and Chisari, 2009; Li Q. et al., 2013) (see below).

Professional bystander plasmacytoid dendritic cells (pDCs) can also secrete type I and type III IFNs after contacting HCV-infected cells (Stone et al., 2013). Infected cells release exosomes containing HCV-RNA that are sensed by the TLR7 receptor of the pDCs (Takahashi et al., 2010; Dreux et al., 2012; Nakai et al., 2015). Interestingly, pDCs respond to viral RNA even though they are not infected by HCV and they do not allow viral replication.

### IFN Signaling Pathway

IFNα receptors (IFNAR), present on the surface of all cell types, bind to type I IFN (IFNαs, IFNβ, and others) (**Figure 2**). Less promiscuous are IFNλ receptors (IFNLR), which bind type III IFN or IFNλ and have a strong influence on HCV replication (Ge et al., 2009; Thomas et al., 2009; Boisvert and Shoukry, 2016). IFN receptor binding to infected and non-infected neighboring cells activates the Janus kinase-signal transducer and activator of transcription (JAK-STAT) pathway. JAK and Tyk2 kinases phosphorylate STAT molecules, STAT1 dimerizes with STAT2 and the heterodimer binds IRF9 to form the IFN-stimulated gene factor 3 (ISGF3). ISGF3 is translocated to the nucleus where it binds the IFN-stimulated response elements (ISREs) present in the ISG promoters to induce their transcription (Hertzog and Williams, 2013; Ivashkiv and Donlin, 2014; Schneider et al., 2014).

### Production of ISGs Affecting HCV Infection

ISGs can be divided into three groups according to their function: (i) to increase the antiviral response; (ii) to inhibit viral replication and (iii) to help cells return to homeostasis. PKR, OAS or RNaseL, MxA, STAT1, STAT2, IRF1, 3, 7, and 9 are ISGs that belong to the first group. They function as positive regulators to reinforce pathogen detection, IFN synthesis and/or signaling and increase ISG expression. Oligoadenylate synthetase (OAS) recognizes PAMPs and activates latent endoribonuclease (RNase L) that cleaves viral and cellular ssRNAs reinforcing the antiviral response and providing new PAMPs (Schneider et al., 2014). Myxovirus resistance-A (MxA) stimulates the production of IFNα and IFNβ and enhances the JAK/STAT pathway (Shi et al., 2017). IRF1 is a transcription factor that binds ISRE-like sequences present in the promoter of several ISGs and induces ISG transcription after the first wave of IFN response (Schoggins et al., 2011).

Other ISGs affect several steps of the HCV life cycle: viral entry or uncoating, viral RNA replication, translation and stability or viral release (Schneider et al., 2014). IFITM proteins (IFN-induced transmembrane proteins) block viral entry by interacting with the CD81 receptor (IFITM1) or by capturing the virions at the endosomes for their degradation (IFITM2 and IFITM3) (Perreira et al., 2013; Narayana et al., 2015). IFIT proteins (IFN-induced proteins with tetratricopeptide repeats) inhibit virus replication and protein translation by affecting eIF3 (Zhou et al., 2013). TRIM proteins (tripartite-motif-containing proteins) affect infection of several viruses at different steps. TRIM5α binds viral capsids to accelerate viral disassembly while TRIM22 inhibits viral transcription and regulation (Hattlmann et al., 2012; Schneider et al., 2014). TRIM22 ubiquitinates NS5A viral protein, interrupting replication in HCV-infected cells (Yang et al., 2016). Viperin/RSAD binds to NS5A to affect virus replication and assembly (Helbig et al., 2005, 2011). Tetherin/BST2 affects HCV release and assembly. Tetherin is known to anchor HIV-1 virions to the plasma membrane, blocking the release of the viral particle (Perez-Caballero et al., 2009). In HCV-infected cells the effect of viperin is milder than in HIV-1 (Dafa-Berger et al., 2012; Pan et al., 2013). Finally, the function of several ISGs is to help the IFN-induced cell to return to homeostasis (Malireddi and Kanneganti, 2013; Chen et al., 2017). Interestingly, as these ISGs decrease IFN signaling, they have proviral effects.

### HCV Evasion from Immune System

Given the potent antiviral properties of ISGs, it is not surprising that HCV has evolved to hide from cellular sensors, block IFN induction and interfere with the action of several antiviral factors. This is achieved by several viral proteins and by the HCV-mediated induction of cellular ISGs that function as negative regulators of the IFN pathway (Arnaud et al., 2010; Imran et al., 2012; Horner and Gale, 2013; Oshiumi et al., 2013a; Gokhale et al., 2014; Yang and Zhu, 2015).

NS3-NS4A is one of the most relevant factors in the HCV evasion strategy. The protease interferes with the signaling induced by the major HCV sensors by cleaving and inactivating MAVS, TRIF, a TLR3 signaling adaptor protein, and Riplet E3 ubiquitin ligase, required for RIG-I ubiquitination, binding to TRIM25 and activation (Foy et al., 2005; Li et al., 2005; Meylan et al., 2005; Oshiumi et al., 2010; Gokhale et al., 2014). Other relevant viral factors are NS4A/B, NS5A and the core proteins, which function by inhibiting the transport of MHC Class I molecules to the cell surface and by blocking the STAT pathway. Core upregulates the protein phosphatase PP2Ac or SOCS3, leading to reduced STAT1 phosphorylation and IFN signaling blockade (Bode et al., 2003; Duong et al., 2004).

Interestingly, several factors with a proven antiviral role against different viruses favor HCV replication. These include the non-canonical sensors PKR and DDX3X. As mentioned above, these factors help IFN synthesis and therefore have an antiviral function. However, induction of IKKα by DDX3X induces the expression of lipogenic genes required by HCV for nucleocapsid assembly (Fullam and Schröder, 2013; Li Q. et al., 2013; Pène et al., 2015). PKR blocks cap-dependent translation of viral proteins by eIF2α phosphorylation. Remarkably, in the case of HCV, the IRES does not require eIF2α for translation (Garaigorta and Chisari, 2009). Instead, PKR blocks translation of transcripts induced soon after viral infection such as antiviral ISGs. Then, in HCV infection PKR blocks translation of cellular antiviral factors while it does not affect the translation of viral proteins.

ISG15 is another general antiviral factor that promotes HCV replication. ISG15 is an ubiquitin-like molecule that can be attached to proteins covalently. This ISGylation occurs co-transcriptionally. Therefore, after HCV infection, the IFN pathway induces ISG15 and the ISGylation machinery, which modifies newly synthesized proteins such as viral proteins and ISGs. IRF3, PKR, MxA, Stat1, Jak1, or RIG-I can be modified by ISGylation. Protein ISGylation changes protein structure and stability, affecting functionality. RIG-I ISGylation blocks RIG-I ubiquitination and functionality. This increases HCV replication by limiting IFN production (Kim et al., 2008; Broering et al., 2010).

Several ISGs are induced by IFN that allow the cell to return to homeostasis and therefore may function as proviral factors. Good examples are ubiquitin specific peptidase 18 (USP18), a protein inhibitor of activated STATs (PIAS) and a suppressor of cytokine signaling (SOCS). USP18 (UBP43) is a protease that displaces ISG15 from its targets and blocks IFN signaling by binding to the IFNAR2 receptor and interfering with JAK binding (Malakhova et al., 2006). The SUMO E3 ligase protein PIAS1 interacts with STAT1 or IRF3 and avoids their binding to DNA, decreasing the STAT1- or IRF3-induced IFN response (Liu et al., 1998; Li R. et al., 2013). SOCS1 and SOCS3 bind the JAK proteins or the IFN receptors and inhibit JAK activity and STAT binding (Yoshimura et al., 2007).

Similar to proteins, several miRNAs and lncRNAs are regulated during infection to promote or repress the antiviral pathway, exerting antiviral or proviral functions.

## Long Non-Coding RNAs (lncRNAs)

Long non-coding RNAs are non-protein coding transcripts longer that 200 nucleotides. Similarly to mRNAs, most lncRNAs are transcribed from RNA polymerase II and are capped at the 5′ end, spliced and polyadenylated (Birney et al., 2007; Guttman et al., 2009). Compared to mRNAs, most lncRNAs are expressed at lower levels, are more cell type-specific and localize preferentially to the nucleus (Djebali et al., 2012). LncRNAs genes are more numerous that coding genes (Iyer et al., 2015). Re-annotation of the cell transcriptome indicates that the human genome may contain more than 90.000 genes, being ∼60.000 of them lncRNA genes.

**FIGURE 3:**
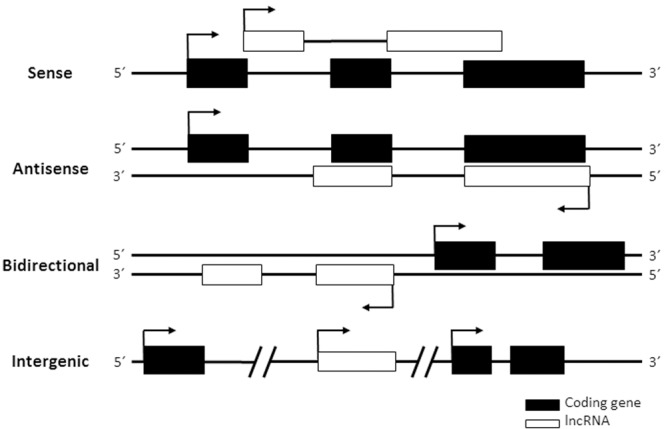
Classification of lncRNAs. Arrows indicate transcription initiation.

Long non-coding RNAs are not easy to classify according to functionality, as the function of most of them is unknown. Instead, most lncRNA classifications are based on genomic localization compared to neighboring genes (Garitano-Trojaola et al., 2013). According to their location in the genome lncRNAs can be grouped into: (i) sense lncRNAs, those that overlap with one or more exons from another transcript in the same strand; (ii) antisense lncRNAs, those that overlap with one or more exons from another transcript in the opposite strand; (iii) bidirectional or divergent lncRNAs, those that share their promoter with another gene in the opposite strand and (iv) intergenic lncRNAs, those that are independent transcripts located between two genes (**Figure 3**). Interestingly, some lncRNAs act as regulators of neighboring genes. Therefore, the genomic location of a given lncRNA gene may help to predict its function. In line with this, enhancer RNAs or eRNAs are lncRNAs transcribed from enhancer regions that participate in enhancing transcription from the promoters of genes located in the same nuclear territory (Orom et al., 2010; Lai et al., 2013; Melo et al., 2013). For function, eRNAs and other lncRNAs need to act in *cis*, at their site of transcription. Instead, *trans-*acting lncRNAs function far from their site of synthesis. Both *cis* and *trans*-acting RNAs can regulate transcription by different mechanisms, including modifying epigenetic regulators and chromatin remodelers, and/or regulating transcription initiation or elongation. Other functions of lncRNAs in the nucleus are as splicing regulators or organizers of subnuclear structures (Tripathi et al., 2010; Mao et al., 2011). In the cytoplasm some may regulate mRNA translation and stability, protein transport or post-translational modifications (Willingham et al., 2005; Cesana et al., 2011; Carrieri et al., 2012; Yoon et al., 2012; Wang et al., 2014). As the function of most lncRNAs is unknown, it is expected that novel functions of lncRNAs will be discovered in the future. Functionality of lncRNAs normally requires a linear sequence or a secondary or tertiary structure that binds to DNA, proteins or other RNAs to form functional complexes. Thus, compared to mRNAs, lncRNAs tend to have structures with higher folding energies (Kertesz et al., 2010).

Over the last decade, lncRNAs have been proved to be key regulators of most cell processes, including cell proliferation, development or cell return to homeostasis (Li X. et al., 2013; Li J. et al., 2016). Therefore, lncRNAs are implicated in all kinds of diseases, including cancer (Prensner and Chinnaiyan, 2011; Wapinski and Chang, 2011; Harries, 2012; Spizzo et al., 2012; Hu et al., 2016). Further, lncRNAs are highly targeted by evolution, suggesting that they may play a relevant role in those processes with a strong evolutionary pressure (Ulitsky et al., 2011; Cech and Steitz, 2014; Marques and Ponting, 2014). One of the drivers of evolution is the antiviral response. In fact, we and other groups have shown that the expression of several lncRNAs is deregulated in response to viral infection.

### LncRNAs in Viral Infection

Viral infections induce strong modifications in the coding and non-coding transcriptome of the infected cell. Infected cells may deregulate cellular lncRNAs and may express viral lncRNAs or chimeric lncRNAs formed by viral and cellular sequences. Many viruses have been shown to express viral lncRNAs. In fact, there are some plant pathogens (viroids and virusoids) whose genome is a lncRNA with replication capacity (Gago-Zachert, 2016; Shimura and Masuta, 2016). Animal viruses such as adenovirus, Epstein–Bar virus, HIV, cytomegaloviruses, flaviviruses, and herpesviruses encode non-coding RNAs that function to affect transcription and RNA stability, and control RNA interference, the antiviral response, the energy of the infected cell and viral pathogenesis (Fortes and Morris, 2016). Interestingly, integration of the HBV genome in repetitive sequences of the genome of the infected cell produces fusions of the Hepatitis B X protein with host LINE1 sequences. These fusion transcripts, named HBx-LINE1, are found in many HBV-derived HCCs and have oncogenic effects (Lau et al., 2014; Moyo et al., 2016).

Cellular lncRNAs may be deregulated by viral proteins, in response to viral replication or in response to the antiviral pathways induced by infection. X protein from HBV decreases the tumor suppressor lncRNA Dreh (Huang et al., 2013), while the Nef protein from HIV decreases lncRNA NRON, leading to increased NFAT and transcription of viral genes (Imam et al., 2015; Lazar et al., 2016). Activation of TLR3, TLR4, or other canonical and non-canonical PAMP sensors and treatment with IFN or TNFα (which triggers NF-κB) lead to changes in the expression of many lncRNAs that are deregulated in infected cells. Some of them have been shown to function as inducers or repressors of the innate antiviral response. This is the case of the negative regulator of antiviral response lncRNA (NRAV) (Ouyang et al., 2014). At homeostasis NRAV levels are high and silence MxA and IFITM3 expression by modulating H3K4me3 and H3K27me3 marks. Upon infection with influenza virus or other viruses, NRAV levels are downregulated leading to an increase in MxA and IFITM3 expression. Then, overexpression of NRAV in mice produces hypersensitivity to influenza infection (Ouyang et al., 2016).

### LncRNAs in HCV Infection

**FIGURE 4:**
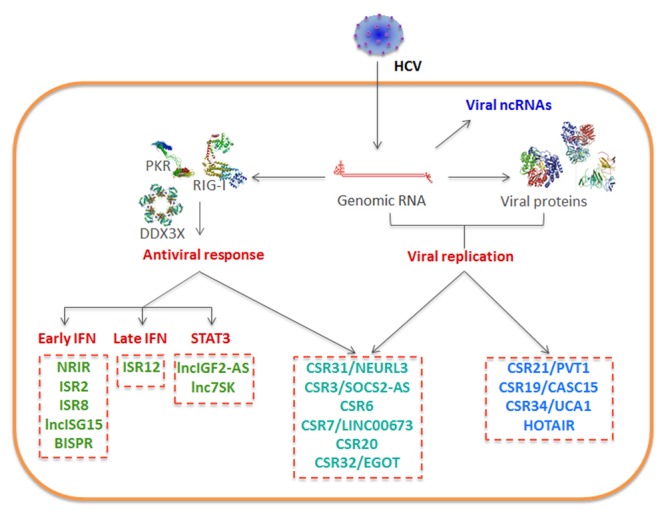
Schematic of lncRNAs deregulated in HCV-infected cells. Viral RNA may be processed to viral ncRNAs. Viral replication, the antiviral response induced by the infection or the combination of both deregulate the levels of several cellular lncRNAs.

Similar to what has been described for other viruses, the lncRNA transcriptome is altered in HCV-infected cells (**Figure 4**). The deregulation of cellular lncRNAs may respond to HCV replication and viral protein expression or to the antiviral response induced against the infection. Further, lncRNAs from the viral genome may also be detected in infected cells.

#### Viral LncRNAs

Transcriptomic analysis of viral RNA in tissue-culture infected cells shows high levels of viral genome enriched in point mutations that generate quasi-species. Major internal deletions or fusions of the viral genome are not detected. Instead, there are HCV subgenomic RNAs that have lost the 5′ end of the IRES sequence, indicating that they could be non-coding RNAs of unknown function. These subgenomic RNAs are generated by the action of XRN1, a cellular exoribonuclease (Moon et al., 2015). XRN1-mediated degradation is not efficient, as miR-122 binding to the 5′ end of the viral RNA protects the genome, leading in part to the essential role played by miR-122 in HCV replication (Thibault et al., 2015).

#### Cellular LncRNAs Induced by the Antiviral Response

In some cases, it is difficult to discriminate whether the deregulation of certain lncRNAs in HCV-infected cells results from the induction of the antiviral response rather than from pure viral replication and viral protein expression (**Figure 4** and **Table 1**). As a general rule, lncRNAs deregulated by the antiviral response are those altered in response to several viruses, different PAMPs and/or IFNs. Transcriptome analysis of cells treated with IFN has allowed the identification of several IFN-stimulated lncRNAs (ISRs) whose levels also increase after HCV infection. They include NRIR, ISR2 (IFN-stimulated lncRNA2), ISR8, lncISG15, and BISPR (BST2 interferon stimulated positive regulator) (Carnero et al., 2014; Kambara et al., 2014, 2015; Barriocanal et al., 2015). Interestingly, all of these are located in the genome very close to ISGs that affect the replication of HCV and other viruses. Thus, NRIR is next to CMPK2 and viperin/RSAD, ISR2 is a neighbor of the GBP cluster, ISR8 is located tail to tail to the IRF1 transcription factor, lncISG15 is very close to ISG15 and BISPR to BST2.

**Table 1:** List of lncRNAs deregulated in HCV-infected cells.

Name	Deregulated in	Inducer	Neighbors	Function	Reference
NRIR	IFN-treated primary hepatocytes, HuH7 and other cultured cells. HCV-infected livers.	Early IFNα	CMPK2, RSAD	Negative regulator of several ISGs (CMPK2, viperin, ISG15, CXCL10, IFIT3, or IFITM1).	Kambara et al., 2014
ISR2	IFN-treated HuH7. HCV-infected livers.	Early IFNα. HCV, SFV, adenovirus, influenza.	GBP cluster	Not known. Predicted to regulate antiviral response.	Carnero et al., 2014
ISR8	IFN-treated HuH7, Hela. HCV-infected livers.	Early IFNα. HCV, SFV, adenovirus, influenza.	IRF1	Not known. Predicted to regulate antiviral response.	Carnero et al., 2014
lncISG15	IFN-treated HuH7. HCV-infected livers.	Early IFNα. HCV, SFV, adenovirus, influenza.	ISG15	Not known	Barriocanal et al., 2015
BISPR	IFN-treated primary hepatocytes, HuH7 and other cultured cells. HCV-infected livers.	Early IFNα, IFNλ and IFNγ. HCV, SFV, adenovirus, influenza.	BST2	Induces BST2 gene expression	Barriocanal et al., 2015; Kambara et al., 2015
ISR12	IFN-treated HuH7	Late IFNα	IL6	Negative regulator of several ISGs as GBP1	Carnero et al., 2014
lncIGF2-AS	HuH7	STAT3	IGF2	Helps HCV replication by regulating PI4P	Xiong et al., 2015
lnc7SK	HuH7	STAT3		Helps HCV replication by regulating PI4P	Xiong et al., 2015
NEURL3/CSR31	HuH7	Antiviral response, HCV, SFV, adenovirus, influenza		Not known	Carnero et al., 2016
SOCS2-AS/CSR3	HuH7, prostate cancer cells	Antiviral response, HCV, SFV, adenovirus, influenza. Androgen signaling.	SOCS2	Depletion of SOCS2-AS1 increases apoptosis-related genes	Carnero et al., 2016
CSR6	HuH7	Antiviral response, HCV, adenovirus		Not known	Carnero et al., 2016
LINC00673/CSR7	HuH7, PDAC and other tumors	Antiviral response, HCV, SFV, adenovirus, influenza. SP1		Studied in several tumors. May function as tumor suppressor or as an oncogene.	Childs et al., 2015; Carnero et al., 2016; Zheng et al., 2016; Huang et al., 2017; Ma et al., 2017; Shi et al., 2017; Yu et al., 2017; Yang et al., 2017
LINC01564/CSR20	HuH7	Antiviral response, HCV, influenza. ROS / NRF2		Not known	Carnero et al., 2016
EGOT/CSR32	HuH7, primary eosinophils	Antiviral response, HCV, SFV, influenza. NF-κB.	ITPR1	Proviral factor by inhibiting ISG expression	Wagner et al., 2007; Carnero et al., 2016
PVT1/CSR21	HuH7 and other cultured cells. Several tumors.	HCV. MYC	MYC	Oncogene that positively regulates MYC and increases proliferation	Graham et al., 1985; Higgs et al., 2013; Tseng et al., 2014; Carnero et al., 2016; Cui et al., 2016; Carramusa et al., 2007
CASC15/CSR19	HuH7, neuroblastoma cells	HCV		Acts as tumor suppressor in some tumors	Maris et al., 2008; Russell et al., 2015; Carnero et al., 2016; Xing et al., 2017
UCA1/CSR34	HuH7	HCV. MYC, SP1, and/or HIF1α		Is overexpressed in different tumors and is involved in anti-cancer drug resistance	Carnero et al., 2016; Kamel et al., 2016; Wang H. et al., 2017; Wang Z.-Q. et al., 2017
HOTAIR	HrpG2 and cells from several carcinomas	HCV core		High levels related to tumor invasion, progression, metastasis and poor prognosis. Gene silencing.	Bhan and Mandal, 2015; Li Z.-Q. et al., 2016

*See the text for details.*

The best known so far is NRIR. NRIR is conserved in the mouse and induced by IFNα in primary hepatocytes and several other cells, including primary keratinocytes, HuH7, HeLa, 293 and Jurkat and mouse myoblast C2C12 cells (Kambara et al., 2014). As NRIR accumulates in the nucleus, it was originally hypothesized that it could be induced by IFN to help transcription of its neighboring genes, CMPK2 and viperin. Surprisingly, depletion of NRIR with two independent siRNAs led to increased expression of viperin and CMPK2 (Kambara et al., 2014). Similar results were observed after NRIR depletion in the levels of IFITM1, IFIT3, CXCL10 and ISG15, ISGs located far from NRIR in the genome, but not in other ISG tested. Unexpectedly, these results were observed in both IFN-treated and untreated cells. NRIR affected transcription and not mRNA stability. Therefore, NRIR is the first described lncRNA that acts as a negative regulator of the transcription of certain ISGs (Kambara et al., 2014). At basal levels, NRIR may help to silence NRIR-target ISGs. After IFN induction, NRIR levels may increase to repress transcription of target ISGs and allow the cell to return to homeostasis. It would be very interesting to understand how NRIR can silence transcription of both neighboring and distal genes. As indicated before, the mRNAs of several ISGs are induced in the liver of HCV-infected patients. Therefore it is not surprising that the liver of HCV-infected patients has higher NRIR levels than non-infected controls (Kambara et al., 2014). As NRIR expression should decrease the levels of HCV antiviral factors such as IFITM1 or viperin, it is likely that NRIR benefits viral replication in infected livers. In fact, NRIR is a proviral factor, as depletion of NRIR in tissue culture upregulates transcription of several ISGs and decreases HCV replication.

ISR2, ISR8, lncISG15, and BISPR are induced early after IFN treatment. These lncRNAs are also upregulated in HCV-infected livers and cultured cells, probably, because of the induction of the IFN signaling pathway in these cells (Carnero et al., 2014; Barriocanal et al., 2015). In fact, ISR2, ISR8, lncISG15, and BISPR are also induced after infection with other viruses. Interestingly, ISR2 and ISR8 are induced more in response to HCV infection than in response to infection with adenovirus, Semliki Forest Virus (SFV) or influenza wild-type or mutant viruses that fail to block the IFN pathway (Carnero et al., 2014). Thus, ISR2 and ISR8 could be induced by HCV also in an IFN-unrelated manner. Although the function of these lncRNAs is not known, ISR2, ISR8, and lncISG15 accumulate preferentially in the cell nucleus, where they could exert regulatory roles. In fact, the levels of these lncRNAs correlate significantly with the levels of their neighboring ISGs, suggesting either that these genes are co-regulated or that the lncRNA could control the expression of the neighboring ISG. In line with this, guilt-by-association studies predict that ISR2 may be involved in the regulation of PAMP receptors and IFN activation and ISR8 may induce the IFN response and the immune system (Carnero et al., 2014).

The role of BISPR has been studied in more detail. BISPR was identified independently by two different groups working with IFN-treated HuH7 cells or primary hepatocytes (Barriocanal et al., 2015; Kambara et al., 2015). BISPR can be induced with IFNα, IFNλ and IFNγ, but not TNFα. Induction with IFNα occurs early after treatment of all cell-lines tested, including primary keratinocytes and hepatocytes, or HuH7, A549, HeLa, Jurkat, or THP1 cell lines. Treatment of the cells with IFN and ruxolitinib, an inhibitor of the JAK/STAT pathway, or transfection of siRNAs that target STAT2 in IFN-treated cells, decreased BISRP levels significantly compared to control cells. Further, the BISPR promoter has conserved binding sequences for STAT1/2/IRF9, IRF1, and IRF7. Together, these results point to BISPR being a bona fide ISG. In fact, as BISPR and BST2/tetherin are located close by in a head-to-head orientation, the BISPR promoter could work as a bidirectional promoter. Not surprisingly, there is a significant positive correlation between the expression of BISPR and BST2/tetherin (Barriocanal et al., 2015; Kambara et al., 2015). Depletion of BISPR with siRNAs in IFN-treated cells leads to decreased levels of BST2. Unlike what has been shown before for NRIR, downregulation of BISPR does not affect the levels of any of the other ISGs evaluated. The molecular mechanism of BISPR action is unknown. However, experiments performed to date are consistent with the following hypothesis: the IFN pathway induces first the expression of BISPR, which is retained in the nucleus and acts *in trans* to induce transcription of BST2/tetherin probably by modulating the effect of the Polycomb complex PRC2 (Kambara et al., 2015). Thus, (i) BISPR induction after IFN-treatment precedes induction of BST2, (ii) BISPR accumulates preferentially in the nucleus of the cell, (iii) overexpression of BISPR increases BST2 levels, suggesting that BISPR does not require to be expressed from a specific genomic position for functionality, (iv) stability of BST2 is not affected after BISPR deregulation, indicating that regulation is at the level of transcription and (v) downregulation of the functional subunit of the PRC2 silencing complex, EZH2, leads to BST2 upregulation, suggesting that BISPR could function by counteracting the suppressive action of PRC2 (Kambara et al., 2015). As BISPR is a positive regulator of BST2, involved in impeding virion release, increased expression of BISPR by IFN could lead to decreased budding of HCV viral particles (Neil et al., 2008; Dafa-Berger et al., 2012; Barriocanal et al., 2015; Kambara et al., 2015). Thus, induction of IFN-induced lncRNAs may be antiviral (as in the case of BISPR) or proviral (as in the case of NRIR) for HCV replication.

Finally, lncRNAs could be induced by a secondary pathway activated by IFN signaling. This is the case of ISR12, a lncRNA that is induced at late times post-IFN treatment and that accumulates preferentially in the cell nucleus (Carnero et al., 2014). ISR12 is located close to IL6, an important pro-inflammatory cytokine. However, the levels of ISR12 and IL6 do not correlate, indicating that IL6 is not regulated by ISR12. In turn, ISR12 depletion with two independent siRNAs leads to increased expression of several ISGs, including GBP1 (Carnero and Fortes, 2016). Thus, as was the case with NRIR, ISR12 is a negative regulator of the expression of ISGs located far away in the genome which could act as a proviral gene. However, ISR12 is not significantly induced in the liver of patients infected with HCV, compared to non-infected patients.

IFN induces STAT3, which functions as a negative regulator of the type I IFN pathway (Wang et al., 2011). STAT3 is a transcription factor induced by stress, growth factors, IL6 and other cytokines. STAT3 can also be induced by HCV infection, as expression of core and other viral proteins induces ROS (Yoshida et al., 2002; Waris et al., 2005; Machida et al., 2006). Then, STAT3 induces lncIGF2-AS and lnc7SK, whose inhibition blocks viral replication by decreasing PI4KIIIalpha, required for MW reorganization (Xiong et al., 2015). Therefore, STAT3 can help viral replication by blocking the IFN pathway and by upregulating lncRNAs that favor MW formation.

#### Cellular LncRNAs Induced by the Antiviral Response and HCV Infection

Among IFN-induced lncRNAs, those that are induced mildly by IFN or PAMPs and strongly by HCV infection deserve special mention (Carnero et al., 2016) (**Figure 4** and **Table 1**). NEURL3, described as a pseudogene, belongs to this group. NEURL3 is induced 12 fold by IFNα, 31 fold after transfection with the dsRNA analog pI:C and almost 2000 fold after HCV infection. Therefore, NEURL3 may be classified as a transcript induced by the antiviral response and other signaling pathways activated in HCV-infected cells. NEURL3 and other lncRNAs that belong to this group were identified after comparing the transcriptome of control cells, cells infected with HCV, cells treated with IFNα and cells that were first infected and then treated with IFNα. LncRNAs induced after HCV infection were called CSRs, from HCV stimulated RNAs (Carnero et al., 2016). CSR3, CSR6, CSR7, CSR20, CSR31/NEURL3, and CSR32 were significantly induced after treatment with pI:C and 4–130 fold more, in cells infected with HCV.

Infection with other viruses also induces the expression of these CSRs, although to milder levels than infection with HCV. CSR3, 7, and 31 are induced after infection with adenovirus, influenza, SFV and HCV, preferentially, with mutant versions that fail to block the IFN pathway; CSR6 is only induced in response to adenovirus, and HCV; CSR20 in response to influenza virus and HCV and CSR32 in response to all RNA viruses tested (HCV, influenza, SFV) but not in response to DNA viruses (adenovirus or HBV) (Carnero et al., 2016). Little is known about the function of these lncRNAs, in relation with HCV infection, with the exception of CSR32/EGOT.

CSR32/EGOT (eosinophil granule ontogeny transcript) is a lncRNA present in all placental mammals that contains several evolutionary conserved and thermodynamically stable secondary structures (Rose and Stadler, 2011). EGOT was first described as a lncRNA preferentially expressed in mature eosinophils where it serves to regulate the expression of toxic eosinophil proteins (Wagner et al., 2007). However, GTEx analysis of the human transcriptome indicates that EGOT is preferentially expressed in the breast and vagina, pancreas, pituitary and kidney cortex, while TCGA analysis shows that EGOT is upregulated in liver, lung and thyroid carcinomas and downregulated in prostate, breast and kidney tumors, where low levels correlate with poor prognosis (Lonsdale et al., 2013; Li J. et al., 2015). Later, it was shown that EGOT is a polyadenylated non-coding RNA induced after infection with HCV in cell lines and in the liver of HCV-infected patients. Interestingly, inhibition of EGOT in HCV-infected cells results in decreased viral genomes, viral titers, and viral proteins (Carnero et al., 2016). Similar results are observed when EGOT is depleted in cells infected with SFV. These results indicate that EGOT is a proviral factor. The proviral function results from EGOT-mediated downregulation of ISGs. This has been observed in massive guilt-by-association studies, where EGOT levels correlate negatively with the levels of genes related to the immune response (Carnero et al., 2016). Further, inhibition of EGOT leads to increased levels of several ISGs including GBP1, ISG15, Mx1, BST2, ISG56, IFI6 and IFITM1, some of which have already been described as blocking HCV or SFV entry, replication or release (Landis et al., 1998; Itsui et al., 2009; Raychoudhuri et al., 2011; Wilkins et al., 2013; Amet et al., 2014; Ooi et al., 2015). Surprisingly, EGOT is a proviral lncRNA induced in response to the antiviral response. Results published so far are in agreement with the following: (i) HCV viral RNA is sensed in the cytoplasm a few hours after infection, as EGOT increases significantly in cells infected with HCV for 5 h or with UV inactivated viruses unable to replicate; (ii) then, viral replication increases EGOT levels further, up to 800 fold, as EGOT decreases in infected cells when replication is inhibited with HCV antivirals; (iii) incoming or replicating viral RNA is sensed by RIG-I and the non-canonical sensor PKR; (iv) these sensors induce transcription by IRF3 and NF-κB; (v) NF-κB is involved in EGOT induction, as induction of the NF-κB pathway with TNFα also results in increased levels of EGOT; (vi) when viral proteins are translated, they block the IFN signaling and synthesis pathways, but they induce NF-κB, which maintains high EGOT levels, (vii) EGOT is a lncRNA expressed from an enhancer region, as the EGOT genomic region has a high ratio of histone 3 lysine 4 monomethylation versus trimethylation (Heintzman et al., 2007), (viii) EGOT decreases the expression of ISGs, which benefits viral replication, encapsidation, and release (Carnero et al., 2016).

As is the case with CSR32/EGOT, which is induced by the antiviral and the NF-κB pathways, other CSRs are induced by other signaling routes activated after HCV infection. HCV infection activates ROS-dependent and independent mechanisms that activate NRF2, which, in turn, upregulates CSR20/RP11-345L23.1/LINC01564 (Burdette et al., 2010; Ivanov et al., 2011; Ashouri et al., 2016). HCV also induces androgen signaling and the transcription factor SP1, which activates transcription of CSR3/SOCS2-AS1 and CSR7/LINC00673, respectively (Kanda et al., 2008; Xiang et al., 2010; Misawa et al., 2016; Zheng et al., 2016).

CSR3/SOCS2-AS1, located in the genome antisense to the Suppressor of Cytokine Signaling-2 (SOCS2) gene, is induced by androgen receptor signaling in prostate cancer cells and promotes androgen-dependent cell growth (Misawa et al., 2016). Depletion of SOCS2-AS1 increases apoptosis-related genes by modulating the epigenetic control of target genes of the androgen receptor signaling pathway.

CSR7/LINC00673 has been studied in several tumors. In human pancreatic ductal adenocarcinoma (PDAC), LINC00673 was significantly downregulated compared to peritumoral tissue. Low expression of the lncRNA correlated with higher occurrence of metastasis, poor differentiation and poor survival. Decreased levels of the lncRNA were shown to promote cell proliferation by repressing the homeobox HNF1A. These results indicate that LINC00673 could be a tumor suppressor (Yang et al., 2017). In fact, detailed studies show that a mutation in the region of LINC00673 is associated with susceptibility to pancreatic cancer (Childs et al., 2015). A G to A change in exon 4 creates a target site for miR-1231 and decreased levels of LINC00673. Then, LINC00673 does not favor the binding between the E3 ubiquitin ligase PRPF19 and PTPN11 and PTPN11 is not ubiquitinated and degraded by the proteasome. High levels of PTPN11 increase SRC-ERK oncogenic signaling and decrease the STAT1 antitumor response, leading to increased risk of tumorigenesis (Zheng et al., 2016). However, LINC00673 has also been described as significantly upregulated in gastric, non-small cell lung cancer and tongue squamous cell carcinomas, where the levels correlate with poor prognosis (Shi X. et al., 2016; Huang et al., 2017; Ma et al., 2017; Yu et al., 2017). In cells derived from these tumors, downregulation of LINC00673 results in decreased cell proliferation, invasion and migration and increased apoptosis. In non-small cell lung cancer and gastric cancer cells, overexpression of LINC00673 has the opposite effect, indicating that this lncRNA is working *in trans* (Shi X. et al., 2016; Huang et al., 2017; Ma et al., 2017). In these cells, it has been shown that LINC00673 works by interacting with the epigenetic regulators LSD1 and EZH2. RNA immunoprecipitation, RNA pull down and ChIP experiments performed with gastric cancer cells show that LINC00673 binds to LSD1 and the Polycomb essential factor EZH2 and induces repression of KLF2 and LATS2. In non-small cell lung cancer cells binding of LINC00673 to EZH2 decreases transcription of HOXA5, a tumor suppressor that blocks metastasis by affecting cytoskeletal remodeling (Ma et al., 2017).

In summary, several lncRNAs are induced in HCV-infected cells both by IFN signaling and alternative pathways. Surprisingly, most of them play important roles in the proliferation and migration of different tumors. Then, we believe that these oncogenic lncRNAs could play a role in the higher incidence of HCC observed in patients infected with HCV.

#### Cellular LncRNAs Induced by HCV Infection

Similarly, several of the lncRNAs that are induced by HCV infection and not by the infection with other viruses or by the antiviral response have also been described as exerting oncogenic functions (Carnero et al., 2016) (**Figure 4** and **Table 1**). These include CSR21/PVT1, CSR19/CASC15, and CSR34/UCA1. We believe that the proliferating environment induced by the expression of oncogenic lncRNAs may be beneficial for HCV replication. In turn, these lncRNAs could induce cell division and other pro-carcinogenic pathways that may contribute to HCC development in HCV-infected patients. In line with this, it has been described that the X protein of HBV decreases the expression of the tumor suppressor lncRNA DREH (Huang et al., 2013).

Hepatitis C virus infection increases the levels of CSR21/PVT-1 around 10 fold. PVT1 is plasmocytoma variant translocation 1 gene (PVT1), an oncogene described as a site of retroviral insertions in murine T lymphomas. Soon after PVT1 identification it was suggested that PVT1 functions to control the neighboring oncogene MYC (Graham et al., 1985). Later, it was shown that PVT1 is upregulated in other tumors, including HCCs (Cui et al., 2016). In fact, it has been described that Hepatitis B virus genome integrates in the region located between MYC and PVT-1 in 12.4% of HCCs that develop early after viral infection and that viral integration correlates with upregulation of MYC and PVT-1, and, most probably, with the development of HCC (Yan et al., 2015). PVT-1 expression is induced by MYC and functions to increase MYC levels, resulting in an oncogenic positive regulatory loop (Carramusa et al., 2007; Tseng et al., 2014). HCV infection could cause increased PVT1 levels by NS5A-mediated induction of MYC (Higgs et al., 2013). Although PVT1’s oncogenic role is closely associated with MYC, the PVT1 gene also encodes for several miRNAs and participates in different DNA rearrangements that lead to aberrant expression and tumorigenesis (Cui et al., 2016). Interestingly, PVT1 transcription also generates a circular RNA that abolishes senescence by sequestering let-7 and allowing accumulation of let7-targetted proliferative genes (Panda et al., 2017).

CSR19/CASC15 increases their levels in HCV-infected cells around sevenfold. CASC15 is a cancer susceptibility candidate with SNPs significantly associated with aggressive neuroblastoma (Maris et al., 2008). Decreased levels of a short isoform of CASC15 associate with poor prognosis and advanced neuroblastoma and downregulation of this isoform increases cell growth and migration (Russell et al., 2015). Similarly, a CASC15 transcript named CANT1 lncRNA functions as a tumor suppressor in Uveal Melanoma (Xing et al., 2017). CANT1 expression reduces tumor formation and metastatic potential by inducing the expression of JPX, FTX, and XIST.

HOX transcript antisense intergenic RNA (HOTAIR) is induced threefold in cells expressing HCV-core protein (Li Z.-Q. et al., 2016). HOTAIR blocks SIRT1 expression by promoter methylation leading to an altered glucose and lipid metabolism that may benefit HCV replication. HOTAIR is upregulated in many tumors, including breast, esophageal, lung, liver and gastric cancers, where high levels of HOTAIR correlate with tumor invasion, progression, metastasis, and poor prognosis. HOTAIR controls cell growth and apoptosis, metastasis, angiogenesis, DNA repair and metabolism by binding key epigenetic regulators such as PRC2 and LSD1 and inducing gene silencing (Bhan and Mandal, 2015).

CSR34/UCA1 is induced in HCV-infected cells over 20 fold, probably by activation of MYC, SP1 and/or HIF1α (Nasimuzzaman et al., 2007; Carnero et al., 2016; Wang H. et al., 2017; Wang Z.-Q. et al., 2017). Reactive oxygen species (ROS) induced by HCV inhibit C/EBPα and stabilize HIF-1α, which are a negative and a positive regulator of UCA1 expression, respectively (Miura et al., 2008; Nishina et al., 2008). Urothelial carcinoma associated 1 (UCA1) has been involved in anti-cancer drug resistance in several tumors (Wang H. et al., 2017). Therefore, UCA1 is overexpressed in different cancers and correlates with poor prognosis. The role of UCA1 in drug resistance is mediated by UCA1 interference with miR27b, miR18a, miR16 and other miRNAs, depending on the tumor cell studied. This increases AKT/mTOR/HIF1a and Wnt/bcatenin signaling which in turn increase MDR1 expression and drug resistance (Wang H. et al., 2017).

UCA1 together with lncRNA WRAP53 are increased in the liver and serum of HCV-derived HCC compared to HCV-derived cirrhotic tissues and healthy livers (Kamel et al., 2016). In fact, several studies have been performed to identify HCV-induced lncRNAs in HCV-related HCCs. The patients chosen had developed liver cirrhosis or HCC in response to HCV infection. In most cases, lncRNA expression profiles of these livers were compared to those of healthy livers. Therefore, it is unclear whether the identified lncRNAs are deregulated by the development of liver cirrhosis or HCC, by HCV infection or by a combination of these factors. In HCV-derived HCCs LINC01419 is upregulated and AF070632 is deregulated (Zhang et al., 2015). However, LINC01419 and other lncRNAs such as ANRIL and HOTTIP are also upregulated compared to adjacent or healthy tissue in both HCV-related and HBV-related HCCs, suggesting that these lncRNAs could be related to all HCCs (Zhang et al., 2015, 2016). Patients with HCV-related hepatic fibrosis show increased levels of the TGFβ-induced lncRNA-ATB compared to healthy controls (Yuan et al., 2014; Fu et al., 2017). Interestingly, LX-2 cells, a line of the fibrosis-inducer hepatic stellate cells, upregulates LncRNA-ATB when incubated with conditional medium from a liver cell expressing HCV core protein. Experiments agree with a model linking HCV infection and liver fibrosis, in which expression of core protein in liver cells induces LncRNA-ATB in hepatic stellate cells, which respond with increased collagen secretion and fibrosis (Fu et al., 2017).

## Concluding Remarks

Taken together, the results published so far, are in agreement regarding the key role of lncRNAs in HCV replication, modulation of the antiviral response and, probably, the development of liver fibrosis and HCC in infected patients. However, further studies are required to identify lncRNAs deregulated at different stages of the viral cycle and of the progression to chronicity, fibrosis, cirrhosis, and HCC. Deregulated lncRNAs should be compared to those deregulated in HCC and cirrhotic livers of different etiologies, including HBV infection and alcohol abuse. Functional studies will help to determine the relevance of the lncRNAs for viral replication and disease progression and the molecular mechanisms involved. Deciphering the bidimensional and tridimensional structure of the RNAs and identifying their DNA, RNA and/or protein-interacting partners will be essential to understand functionality. The results obtained so far allow us to suggest that the study of HCV-related lncRNAs will aid in the identification of lncRNAs that function as proviral or antiviral agents, positive or negative regulators of the immune system, oncogenes, tumor suppressors, metabolic regulators, or profibrogenic factors. Research on these lncRNAs may aid in the development of novel therapies for the treatment of immune and infection diseases and cancer.

## Author Contributions

MB and PF revised all the literature, wrote the review and made the figures.

## Conflict of Interest Statement

The authors declare that the research was conducted in the absence of any commercial or financial relationships that could be construed as a potential conflict of interest.
